# Cytokine Concentrations in Aqueous Humor of Eyes With Circumscribed Choroidal Hemangioma

**DOI:** 10.3389/fendo.2022.916789

**Published:** 2022-06-28

**Authors:** Kailin Liu, Lili Guo, Yong Cheng, Jianhong Liang

**Affiliations:** Beijing Key Laboratory of Diagnosis and Therapy of Retinal and Choroid Diseases, Eye Diseases and Optometry Institute, Department of Ophthalmology and Clinical Centre of Optometry, Peking University People’s Hospital, College of Optometry, Peking University Health Science Center, Beijing, China

**Keywords:** aqueous humor, cytokines, circumscribed choroidal hemangioma, VEGF - vascular endothelial growth factor, IP-10 (IFN-γ Inducible Protein 10)

## Abstract

**Purpose:**

Anti-vascular endothelial growth factor (anti-VEGF) treatment are now widely used in patients with circumscribed choroidal hemangioma (CCH), however the concentrations of VEGF and other cytokines in CCH patients have not been known before. The study was conducted to compare various cytokine concentrations in the aqueous humor of eyes with CCH and control.

**Methods:**

A total of 16 eyes of 16 patients with CCH, and 15 eyes of 15 patients with cataract as the control group were examined. Aqueous humor samples were assessed for 30 angiogenic and inflammatory cytokines by Luminex bead-based multiplex array.

**Results:**

Significantly, compared with control group, higher concentrations of VEGF-A and IP-10 were found in the CCH patients (P = 0.002 and P < 0.001).

**Conclusions:**

VEGF-A and IP-10 might be involved with the angiogenic and antiangiogenic process in CCH patients, which provides new insight into the pathophysiology of CCH and could be potential targets for treatment.

## Introduction

Choroidal hemangioma is a benign vascular tumor which is classified into two types based on the extent of choroidal involvement, circumscribed and diffuse. Circumscribed choroidal hemangioma (CCH) generally arises unilaterally and solitarily without systemic associations (1, 2). Although CCH is a benign tumor, it is still a vision threatening condition because of hyperopic shift or exudative retinal detachment (3). When it becomes symptomatic, treatment modalities include photodynamic therapy, laser photocoagulation, transpupillary thermotherapy, external beam and proton beam radiation (PDT), gamma knife radiosurgery, and plaque brachytherapy (4–9). However, because of complications associated with radiotherapy and the less favorable vision outcome, ophthalmologists seek other treatment options for CCH and start to use anti-vascular endothelial growth factor (anti-VEGF) agents to treat CCH and concomitant exudative retinal detachment. In 2009, Sagong et al. first reported that the use of intravitreal bevacizumab, alone or combined with PDT may be helpful for symptomatic CCH (10). Several studies since then confirmed the effectiveness of intravitreal anti-VEGF agents used in CCH patients (11, 12). However, to our knowledge, there are no evidence showing that the ocular concentration of VEGF actually raises in CCH patients. While VEGF and other cytokines profile in aqueous humor in patients with CCH haven’t been reported before, we aim to investigate both angiogenic and inflammatory cytokines in the aqueous humor among CCH patients and controls by using multiplex immunoassay.

## Methods

The Ethics Committee and Institutional Review Board of Peking University People’s Hospital approved this study, and written informed consent was obtained from each patient in accordance with the Declaration of Helsinki. All the patients were diagnosed at Peking University People’s Hospital. Exclusion criteria included previous intraocular surgery or previous treatment, such as PDT or plaque brachytherapy, concomitant ocular diseases, concomitant systemic diseases such as hypertension or diabetes, prior cardio-cerebrovascular accidents within the past 6 months. Undiluted aqueous humor samples were collected from 16 eyes with CCH before plaque brachytherapy and 15 eyes of cataract patients before cataract surgery. Aqueous samples of 0.05mL from each eye were extracted using a standard sterilization procedure and immediately stored in a freezer at -80°C until assay. And they were tested within 6 months of collection.

Cytokines were measured *via* Luminex X-MAP technology using the Procarta Immunoassay kit (Panomics Inc, Fremont, CA), as described previously (13).

All data were analyzed using SPSS 26.0 for Mac (SPSS, IBM Corp, New York). Data are presented as the mean ± standard deviation, and the normality of the distribution was assessed by the Shapiro–Wilk test. The nonparametric Mann–Whitney rank sum test and *t*-test were performed to compare the differences between the study group and the control group. Two-tailed probability of less than 0.05 was considered to be a statistically significant difference.

## Results

The study included 31 patients, with 16 patients in the study group and 15 patients in the control group. The details for all subjects including sex and age were listed in **Table 1**. Men in CCH group and control group were 11 (68.8%) and 7 (46.7%), respectively. Gender was not significantly different among the groups. The average age of the patients with CCH and control was 35.1 ± 17.6 and 65.2 ± 11.1 years old, respectively. However, age showed statistical difference among 2 groups. CCH patients were significantly younger than patients in the control group (P < 0.001).

**Table 1:** Demographics of study population.

Characteristic	CCH patients	Controls	*P* value
No. of patients	16	15	
Gender (male/female)	11/5	7/8	0.213^*^
Age (mean ± SD)	35.1 ± 17.6	65.2 ± 11.1	< 0.001^†^
Range	11-70	39-83	

Data are shown as mean ± SD or number.

^*^ *X* ^2^ test.

^†^Mann-Whitney U test.

The baseline ocular and tumor characteristics of CCH patients were summarized in **Table 2**. Right eye was involved in 9 patients. The mean largest basal tumor diameter was 11.8 mm (range, 8.2–15.0), and the mean tumor thickness was 3.9mm (range, 2.6-5.9). 10 patients were associated with exudative retinal detachment.

**Table 2:** Summary data on baseline ocular and tumor variables in patients with CCH.

Characteristic	
Eye	
Right	9 (56.3%)
Left	7 (43.7%)
Tumor size	
Base (mean ± SD)	11.8 ± 3.0 (8.2-15.0)
Thickness (mean ± SD)	3.9 ± 1.0 (2.6-5.9)
Exudative retinal detachment	
Yes	10 (62.5%)
No	6 (37.5%)

Data are shown as mean ± SD (range) or number (%).

**Figure 1:**
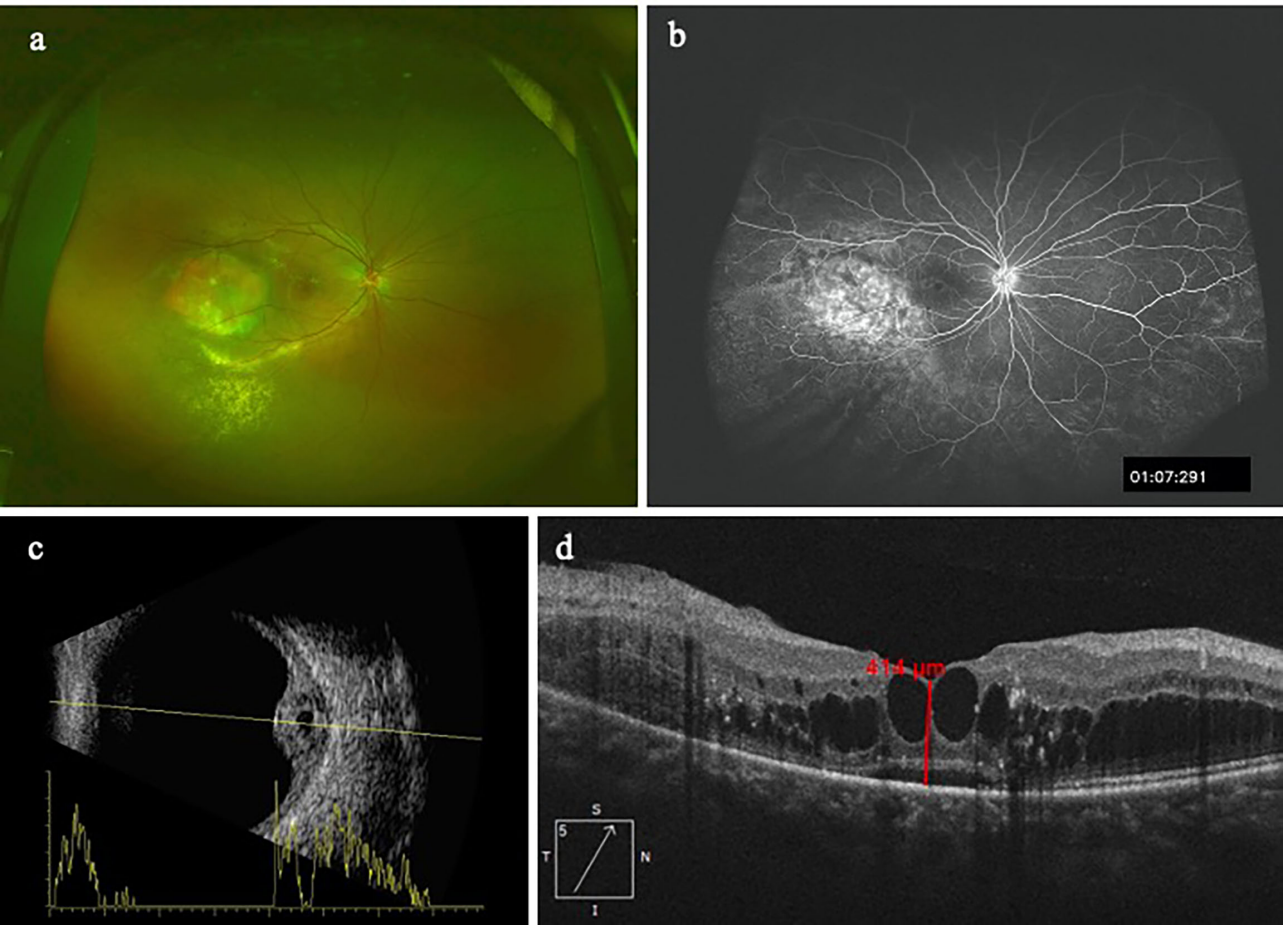
A patient with CCH. The concentration of IP-10 in aqueous humor is 76.4 pg/ml while the concentration of VEGF-A is 987.57pg/ml. **(A)** CCH temporal to the fovea; **(B)** The FFA clearly delineates the hemangioma with leakage; **(C)** Ultrasonography reveals a tumor thickness of 4.3mm; **(D)** OCT image shows intraretinal cystic edema and subretinal fluid.

**Table 3** showed the concentrations of the 30 cytokines in the aqueous humor samples. Because the data on the aqueous concentration of IL-6, IL-8, IL-9, IL-23, IL-27, IL-1α, IL-1RA, TNF-β, Eotaxin, GRO-α, IP-10, RANTES, NGF-β, BDNF, EGF, HGF, LIF, PlGF-1, SCF, VEGF-A did not show a normal distribution according to Shapiro-Wilk test, we performed a Mann-Whitney rank-sum test on the data. Among the 30 cytokines, the aqueous humor levels of IP-10 and VEGF-A in the CCH group were significantly higher than the control group (P = 0.002, and P < 0.001). In contrast, the levels of IL-5, IL-22, IL-9, IL-31, TNF-β, GRO-α, RANTES, NGF-β in the aqueous humor were significantly lower in the CCH group than controls (P = 0.027, P = 0.008, P < 0.001, P = 0.004, P = 0.001, P = 0.002, P = 0.011, P = 0.005, respectively). **Figure 1** showed ocular examinations and cytokine concentrations in a representative CCH patient.

**Table 3:** Aqueous humor concentrations (pg/mL) (mean ± SD) of cytokines in CCH patients and subjects undergoing routine cataract surgery (control group).

Cytokine	CCH patients (pg/ml)	Controls (pg/ml)	*P* value
IL-5	6.87 ± 3.63	10.45 ± 4.86	**0.027**
IL-6*	363.38 ± 630.39	760.3 ± 1297.95	0.545
TNF-α	7.35 ± 4.69	9.49 ± 6.65	0.307
IL-22	18.33 ± 3.96	22.57 ± 4.29	**0.008**
IL-23*	17.1 ± 14.4	16.06 ± 8.98	0.711
IL-27*	11.55 ± 13.49	16.08 ± 25.37	0.984
IL-9*	2.81 ± 2.56	6.66 ± 2.88	**<0.001**
IFN-α	2.34 ± 1.13	2.86 ± 1.27	0.237
IL-31	5.18 ± 3.3	9.07 ± 3.55	**0.004**
IL-1α*	0.86 ± 0.76	1.06 ± 0.65	0.401
IL-1RA*	163.37 ± 112.76	522.88 ± 900.32	0.953
IL-7	4.1 ± 1.5	4.49 ± 1.39	0.460
TNF-β*	9.63 ± 10.58	17.73 ± 7.16	**0.001**
Eotaxin*	3.57 ± 1.64	3.75 ± 2.58	0.599
GRO-α*	1.34 ± 1.96	3.39 ± 2.51	**0.002**
IL-8*	9.56 ± 5.46	15.49 ± 11.21	0.093
IP-10*	114.73 ± 63.29	37.48 ± 43.75	**<0.001**
MCP-1	1372.67 ± 260.18	1329.86 ± 376.34	0.714
MIP-1α	1.54 ± 1.13	2.47 ± 1.87	0.104
MIP-1β	19.84 ± 10.42	25.6 ± 17.88	0.278
SDF-1α	184.19 ± 86.93	130.28 ± 70.14	0.068
RANTES*	0.32 ± 0.31	0.63 ± 0.21	**0.011**
NGF-β*	2.6 ± 0.33	2.89 ± 0.32	**0.005**
BDNF*	0.74 ± 0.57	0.79 ± 0.2	0.423
EGF*	1.57 ± 1.61	2 ± 2.46	0.861
HGF*	885.18 ± 364.99	798.38 ± 617.26	0.318
LIF*	19.22 ± 18.25	10.37 ± 7.68	**0.047**
PlGF-1*	2.45 ± 2.85	1.57 ± 1.54	0.682
SCF*	4.63 ± 2.23	4.83 ± 2.48	1.000
VEGF-A*	1217.93 ± 891.82	300.36 ± 152.61	**<0.001**

Data are shown as mean ± SD or number.

SD, Standard deviation, IL, Interleukin; TNF, tumor necrosis factor; IFNα, interferon-α; GROα, growth-related gene product α; IP-10, interferon inducible protein 10; MCP-1, monocyte chemoattractant protein; MIP-1, macrophage inflammatory protein 1; SDF1-α, stromal-derived factor 1-α; RANTES, regulated upon the activation of normal T cell expressed and secreted; NGF, nerve growth factor; BDNF, brain-derived neurotrophic factor; EGF, epidermal growth factor; HGF, hepatocyte Growth Factor; LIF, leukemia inhibitory factor; PlGF, placental growth factor; SCF, stem cell factor; VEGF, vascular endothelial growth factor. Bold = P value was statistically significant (P < 0.05 was deemed to be statistically significant which using Student t test when data were normal distributed or Mann-Whitney U test when data were unnormal distributed. * means unnormal distribution including IL-6, IL-8, IL-9, IL-23, IL-27, IL-1α, IL-1RA, TNF-β, Eotaxin, GRO-α, IP-10, RANTES, NGF-β, BDNF, EGF, HGF, LIF, PlGF-1, SCF, VEGF-A.

## Discussion

In the present study, we investigated the levels of 30 cytokines in aqueous humor from patients with CCH and controls, and explored whether there were differences among the two groups. Our results revealed that VEGF-A and IP-10 concentrations in aqueous humor of CCH patients were significantly higher than controls. In the meantime, the levels of IL-5, IL-22, IL-9, IL-31, TNF-β, GRO-α, RANTES, NGF-β in the aqueous humor were significantly lower in the CCH group than controls.

VEGF-A is an important pro-angiogenic factor associated with angiogenesis in tumors and retinal vascular disease. Anti-VEGF therapy has been widely used in the treatment of diabetic retinopathy, retinal vein occlusion and neovascular age-related macular degeneration. With regard to management of intraocular tumors, Kenawy et al. reported that intravitreal anti-VEGF therapy could reduce vasoproliferative retinal tumor-associated exudation (14). Ziemssen et al. reported a rapid decrease in exudation after intravitreal bevacizumab in a patient with juxtapapillary retinal capillary hemangioma (15). On the basis of these observations, Sagong et al. first reported 3 cases of CCH effectively managed with intravitreal bevacizumab alone or combined with PDT (10). Since then, Mandal et al. has documented the complete resolution of leakage after intravitreal bevacizumab in 3 cases of CCH patients and Lai et al. showed that using intravitreal conbercept as primary treatment for exudative CCH was effective in maintaining BCVA and decreasing the central foveal thickness (11, 12). Although anti-VEGF therapy has been proved effective in treating CCH with exudative retinal detachment, the current study is the first report which actually shows the elevation of VEGF-A in aqueous humor of from patients with CCH. This provides a reasonable explanation for the effectiveness of intravitreal anti-VEGF treatment in CCH patients. In the meantime, this indicates further studies are needed to observe the changes of VEGF-A before and after treatment in CCH patients, and the aqueous humor level of VEGF-A in treatment-naïve CCH patients may be a prognostic factor of anti-VEGF treatment responses.

Interferon-inducible protein 10 (IP-10) belongs to a group of α-chemokines and possess high affinity for the CXCR3 receptors found on activated T cells and natural killer cells (16). IP-10 is a chemoattractant to Th1 lymphocytes and monocytes and has antiangiogenic and antifibrotic effect (17). The antiangiogenic effect of IP-10 works through the inhibition of proliferation of endothelial cells and the induced apoptosis of them (18). Furthermore, Bodnar *et al.* found that IP-10 could inhibits VEGF-Induced endothelial tube formation (19). Elevation of IP-10 has been reported in uveal melanoma, diabetic Macular Edema, age-related macular degeneration and polypoidal choroidal vasculopathy (20–23). Our results showed that IP-10 also played a role in the pathophysiology in CCH, probably was a biomarker of angiogenic and antiangiogenic process.

The present study had several limitations, including the relatively small sample size, and the possible bias caused by sample selection. Furthermore, the study design did not allow examination and comparison of the changes of the cytokine concentrations in aqueous humor before and after treatment. Further studies with a larger number of the patients and the investigation of changes of cytokine concentrations are crucial for confirming the role of these angiogenesis and inflammation associated cytokines in the pathophysiology of CCH.

## Conclusion

Our research studied the concentrations of 30 cytokines in aqueous humor of patients with CCH and the controls. To the best of our knowledge, the cytokine concentrations in aqueous humor of patients with CCH have not been reported in previous researches. In the current study, we found intraocular levels of VEGF-A and IP-10 significantly elevated in patients with CCH, which may be associated with the pathophysiology of CCH. Importantly, these angiogenic and inflammatory cytokines may be potential novel therapeutic target for CCH.

## Data Availability Statement

The raw data supporting the conclusions of this article will be made available by the authors, without undue reservation.

## Ethics Statement

The studies involving human participants were reviewed and approved by the Ethics Committee and Institutional Review Board of Peking University People’s Hospital. The patients/participants provided their written informed consent to participate in this study.

## Author Contributions

JL and YC contributed to conception and design of the study. KL and LG organized the database. LG performed the statistical analysis. KL wrote the first draft of the manuscript. LG and YC wrote sections of the manuscript. All authors contributed to manuscript revision, read, and approved the submitted version.

## Funding

This study was supported by the National Key R&D Program of China (2020YFC2008200); Beijing Municipal Science and Technology Commission (Grant Number Z191100007619041. The funders had no role in the study design, data collection and analysis, decision to publish or preparation of the manuscript.

## Conflict of Interest

The authors declare that the research was conducted in the absence of any commercial or financial relationships that could be construed as a potential conflict of interest.

## Publisher’s Note

All claims expressed in this article are solely those of the authors and do not necessarily represent those of their affiliated organizations, or those of the publisher, the editors and the reviewers. Any product that may be evaluated in this article, or claim that may be made by its manufacturer, is not guaranteed or endorsed by the publisher.
